# The cytochrome P450 reductase CprA is a rate-limiting factor for Cyp51A-mediated azole resistance in *Aspergillus fumigatus*

**DOI:** 10.1128/aac.00918-23

**Published:** 2023-10-10

**Authors:** Alexander Kühbacher, Petra Merschak, Hubertus Haas, Maximilian Liebl, Christoph Müller, Fabio Gsaller

**Affiliations:** 1 Institute of Molecular Biology, Biocenter, Medical University of Innsbruck, Innsbruck, Austria; 2 Department of Pharmacy, Center for Drug Research, Ludwig-Maximilians Universität München, Munich, Germany; University Children's Hospital, Münster, Münster, Germany

**Keywords:** *Aspergillus fumigatus*, azole resistance, *cyp51A*, sterol C14-demethylase, cytochrome P450 reductase

## Abstract

Azole antifungals remain the “gold standard” therapy for invasive aspergillosis. The world-wide emergence of isolates resistant to this drug class, however, developed into a steadily increasing threat to human health over the past years. In *Aspergillus fumigatus*, major mechanisms of resistance involve increased expression of *cyp51A* encoding one of two isoenzymes targeted by azoles. Yet, the level of resistance caused by *cyp51A* upregulation*,* driven by either clinically relevant tandem repeat mutations within its promoter or the use of high expressing heterologous promoters, is limited. Cytochrome P450 enzymes such as Cyp51A rely on redox partners that provide electrons for their activity. *A. fumigatus* harbors several genes encoding putative candidate proteins including two paralogous cytochrome P450 reductases, CprA and CprB, and the cytochrome *b*_5_ CybE. In this work, we investigated the contribution of each *cprA*, *cprB,* and *cybE* overexpression to *cyp51A*-mediated resistance to different medical and agricultural azoles. Using the bidirectional promoter *PxylP*, we conditionally expressed these genes in combination with *cyp51A*, revealing *cprA* as the main limiting factor. Similar to this approach, we overexpressed *cprA* in an azole-resistant background strain carrying a *cyp51A* allele with TR34 in its promoter, which led to a further increase in its resistance. Employing sterol measurements, we demonstrate an enhanced eburicol turnover during upregulation of either *cprA* or *cyp51A*, which was even more pronounced during their simultaneous overexpression. In summary, our work suggests that mutations leading to increased Cyp51A activity through increased electron supply could be key factors that elevate azole resistance.

## INTRODUCTION

Members of the azole antifungal drug class constitute the preferred option for first-line treatment of invasive aspergillosis (1, 2), an often deadly infection that is mainly caused by the ubiquitous fungal mold pathogen *Aspergillus fumigatus* (3). Four years ago, the Centers for Disease Control and Prevention in the United States put azole-resistant *A. fumigatus* on the watch list for antimicrobial resistance threats, and only recently, this pathogenic mold was listed within the critical group of the World Health Organization’s fungal priority pathogens list (4, 5). The world-wide occurrence and increase in azole-resistant isolates progressively develop into a threat to current medical therapeutic strategies (6, 7). Clinical resistance is mainly connected to mutations of the azole drug target encoding gene *cyp51A* or its promoter. However, a large proportion is attributed to non-*cyp51A*-based resistance mechanisms such as those arising from mutations of genes encoding 3-hydroxy-3-methyl-glutaryl-coenzyme A reductase Hmg1, components of the CCAAT-binding complex, efflux pumps as well as so far uncharacterized mutations (8 – 10).

**Fig 1:**
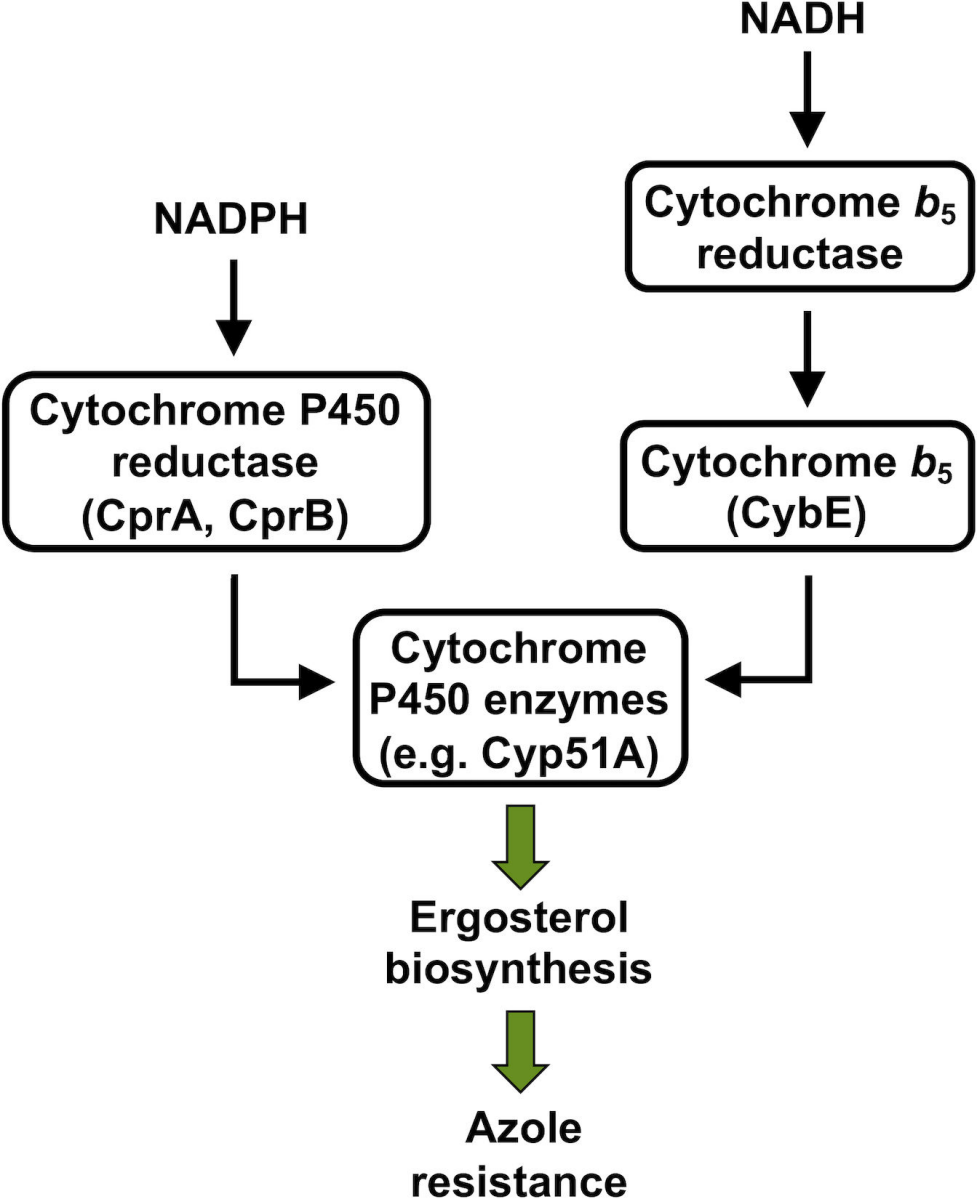
Proposed electron transfer path from NADPH and NADH by cytochrome P450 reductase and cytochrome *b*_5_ to cytochrome P450 enzymes in *A. fumigatus*. Black arrows illustrate the delivery of electrons. Green arrows indicate the consequent positive impact on ergosterol biosynthesis and azole resistance.

Inhibition of Cyp51 by azoles leads to the accumulation of toxic C14-methylated sterols and concomitant reduced ergosterol production (11). TR34/L98H and TR46/Y121F/289A (12 – 15), two of the most common mechanisms of azole resistance found in clinical isolates across the world (9), diminish growth-hampering, antifungal effects by azoles at least, in part, by overexpressing *cyp51A*. TR34 and TR46 (14, 15) as well as the less common TR53 and TR120 (16, 17) illustrate tandem repeats (TRs) in the *cyp51A* promoter that contain duplications of binding sites for its transcriptional activators SrbA and AtrR (18 – 22). *In vitro* studies using recombinant strains demonstrated that increased expression of *cyp51A* caused by TR34, TR46 or high-expressing constitutive promoters, is limited to approximately 2- to 4-fold (12, 13, 15, 23). In this regard, it has to be mentioned that cytochrome P450 enzymes such as sterol C14-demethylase Cyp51A require two electrons for their catalytic activity that are provided by redox partners such as the NADPH cytochrome P450 reductase (CPR) and/or cytochrome *b*_5_, which is part of the cytochrome *b*_5_ reductase (CB5R) system (24, 25) (Fig. 1). Through the NADPH-connected electron transport chain, it was originally proposed that P450 enzymes receive the electrons that are critical for their enzymatic activity *via* two single electron steps from NADPH cytochrome P450 reductase. Cytochrome *b*_5_ was also suggested to provide electrons, however, only the second in this cycle (26). Later, work on Cyp51-based azole sensitivity in yeast revealed cytochrome P450 reductase as the main, but not only factor providing the two electrons for Cyp51 activity (27) as overexpression of the cytochrome *b*_5_ encoding gene *CYB5* could increase ketoconazole resistance in a CPR-deficient background (28). This led to the hypothesis that both necessary electrons for Cyp51 activity can be delivered by the CB5R system, which was confirmed soon after (25). Based on the necessity of sufficient electron supply for P450 enzymatic activity, it is somewhat not surprising that the increase in *cyp51A* transcript or protein content does not fully correlate with the level of resistance. The *A. fumigatus* genome encodes two putative CPRs, CprA (AFUB_077020) and CprB (AFUB_023960), as well as the cytochrome *b*_5_ CybE (AFUB_021740) which together with NADH cytochrome *b*_5_ reductase constitutes the CB5R system in this fungus (29).

In this work, we studied the consequences of overexpression of each component *cprA*, *cprB,* and *cybE* during simultaneous upregulation of *cyp51A* on *A. fumigatus* resistance to triazole-based sterol C14-demethylase inhibitors (further called azoles) of medical (voriconazole, VRZ; itraconazole, ITZ; isavuconazole, ISZ) and agricultural (tebuconazole, TBZ; epoxiconazole, EPZ) importance (1, 30, 31). For the conditional expression mutant displaying the highest degree of azole resistance, sterol measurements were carried out to monitor the impact of its increased activity on the turnover of eburicol, substrate of Cyp51A, as well as ergosterol. We further conditionally expressed the candidate in a recipient strain carrying a *cyp51A* allele with TR34 in its promoter, to assess the consequences of increased activity of the Cyp51A redox partner during TR-driven upregulation of *cyp51A*.

## RESULTS

### Combined overexpression of *cyp51A* and *cprA* leads to high levels of azole resistance

**Fig 2:**
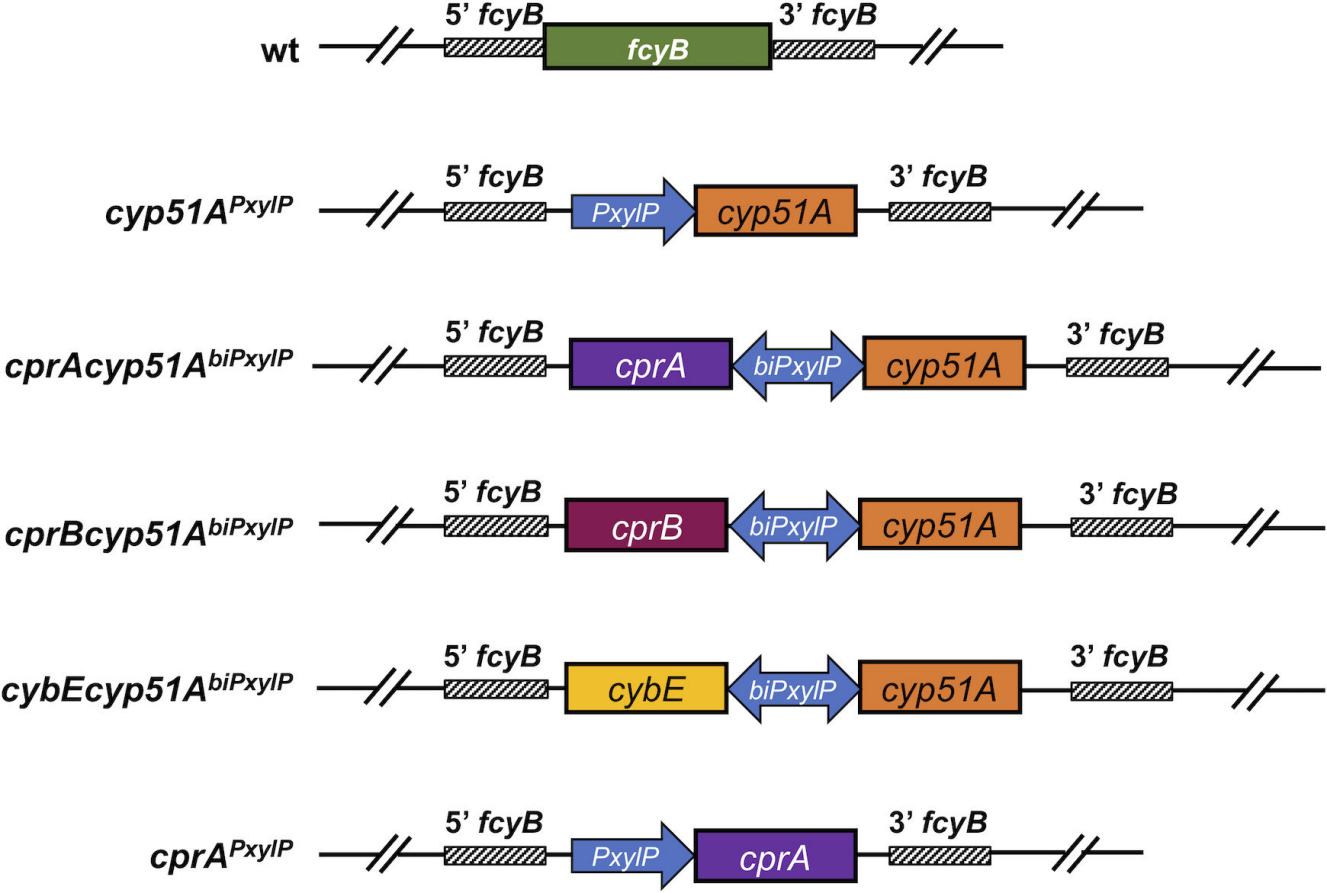
Scheme of strains carrying tunable gene variants of *cyp51A* and its potential redox partners. Constructs comprising gene cassettes with *PxylP*-driven *cyp51A* (*cyp51A^PxylP^*) and *cprA* (*cprA^PxylP^*) as well as *cyp51A* in combination with *cprA* (*cprAcyp51A^biPxylP^*), *cprB* (*cprBcyp51A^biPxylP^*), and *cybE* (*cybEcyp51A^biPxylP^*) under bidirectional (*biPxylP*) control of *PxylP* (35) were site-directed integrated at the counterselectable marker locus *fcyB* (36).

Similar to the clinically relevant tandem repeat mutations found in TR34/L98H and TR46/Y121F/T289A (12, 13, 15), it has been demonstrated that overexpression of *cyp51A* increases resistance to azoles, however, only to a certain extent (23). Based on previous work that focused on CPR and cytochrome *b*_5_ (25, 27 – 29, 32, 33), we speculated that a shortage of Cyp51A redox partners could be rate-limiting in this scenario. To test this hypothesis, we overexpressed *cyp51A* (strain *cyp51A^PxylP^*) together with *cprA* (strain *cprAcyp51A^biPxylP^*) and *cprB* (strain *cprBcyp51A^biPxylP^*) as well as *cybE* (strain *cybEcyp51A^biPxylP^*), utilizing the bidirectional, xylose-inducible promoter *PxylP* (34, 35). Susceptibilities of strains (Fig. 2) to different azoles were monitored during non-inducing (−xylose) and inducing (+xylose) conditions (Table 1). In comparison to wild type (wt), overexpression of *cyp51A* on its own raised minimum inhibitory concentration (MIC) levels 2- to 4-fold to VRZ, ITZ, ISZ, TBZ, and EPZ. While increased expression of *cprB* and *cybE* together with *cyp51A* elevated resistance only marginally in comparison with *cyp51A^PxylP^* only, concomitant upregulation of *cyp51A* and *cprA* led to a ≥8-fold increase in resistance to all compounds tested. To assess the influence of increased *cprA* expression on its own, we generated the strain *cprA^PxylP^* carrying a *PxylP*-driven *cprA* copy. Upregulation of *cprA* elevated MICs similar to that observed for induction of *cyp51A* only.

**TABLE 1:** MIC values of strains carrying tunable gene variants of *cyp51A* as well as its potential redox partners^*a*^

	VRZ MIC (µg/mL)		ITZ MIC (µg/mL)		ISZ MIC (µg/mL)		TBZ MIC (µg/mL)		EPZ MIC (µg/mL)	
	**−xyl**	**+ xyl**	**−xyl**	**+ xyl**	**−xyl**	**+ xyl**	**−xyl**	**+ xyl**	**−xyl**	**+ xyl**
A1160P+ (wt)	0.25	0.25	0.5	0.5	0.5	0,5	1	1	4	4
*cyp51A^PxylP^*	0.25	0.5–1	0.5	1	0.5	1	1	4	4	16
*cprAcyp51A^biPxylP^*	0.25	2–4	0.5	>16	0.5	4	1	16	4	>16
*cprBcyp51A^biPxylP^*	0.25	1	0.5	1	0.5	1	1	4	4	16
*cybEcyp51A^biPxylP^*	0.25	1	0.5	1	0.5	1	1	4	4	16
*cprA^PxylP^*	0.25	0.5–1	0.5	1	0.5	1	1	4	4	16

^*a*^ Voriconazole (VRZ), itraconazole (ITZ), isavuconazole (ISZ), tebuconazole (TBZ), and epoxiconazole (EPZ) susceptibilities of strains were analyzed following the broth microdilution method according to EUCAST (37). Strains were grown in the presence (+xyl) and absence (−xyl) of 1% xylose.

### Overexpression of *cprA* leads to increased eburicol turnover and elevates ergosterol levels during voriconazole treatment

**Fig 3:**
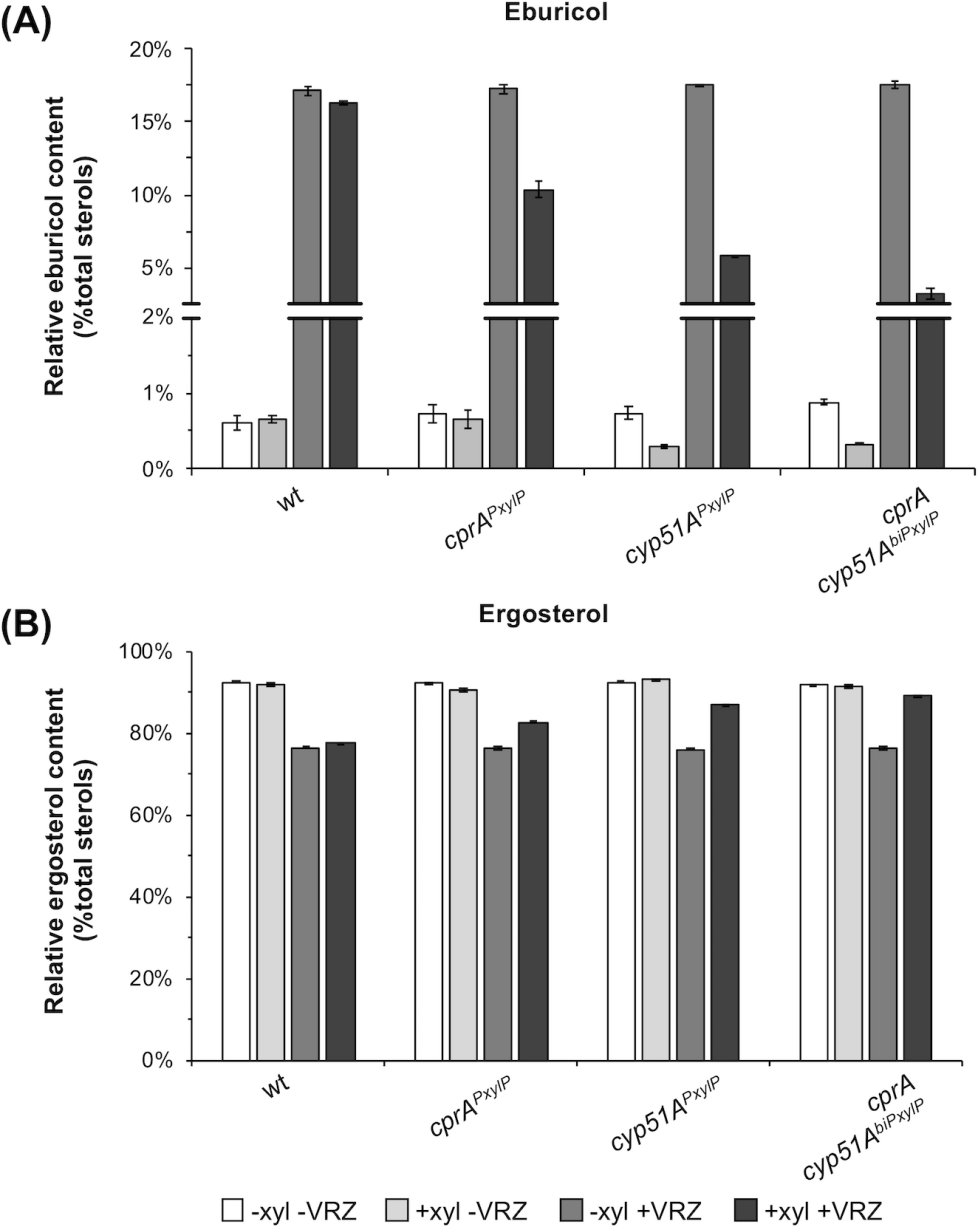
Relative amounts of (A) eburicol and (B) ergosterol in conditional expression strains in the presence (+VRZ) and absence of voriconazole (−VRZ). Strains were grown in liquid AMM during inducing (+xyl) and non-inducing (−xyl) conditions. Error bars indicate the standard deviation of the respective samples.

The high level of resistance of the strain carrying *cprAcyp51A^biPxylP^* suggests that this strain harbors significantly increased Cyp51A activity during *PxylP*-inducing conditions. To monitor the corresponding effects on sterol C14-demethylation and ergosterol biosynthesis, we analyzed the impact of individual and combined overexpression of *cyp51A* and *cprA* on the sterol pattern with focus on the turnover of the Cyp51A substrate eburicol and the final product ergosterol, in the presence and absence of VRZ which is employed as a first-line treatment against invasive aspergillosis (1). Under non-inducing conditions, eburicol and ergosterol levels in wt were 0.6% and 92.4% relative to the total sterol content, respectively (Fig. 3, for details, see Table S3). VRZ exposure led to an increase of eburicol to 17.1% and reduction of ergosterol to 76.5%. Similar levels of eburicol (17.1%–17.6%) and ergosterol (76.0%–76.5%) were detected in the tunable mutant strains without induction. In agreement with their potential contribution to Cyp51A-mediated sterol C14-demethylation, induction of either *cprA* or *cyp51A* decreased the eburicol content (*cprA^PxylP^*: 10.4%; *cyp51A^PxylP^*: 5.8%) and increased ergosterol levels (*cprA^PxylP^*: 82.7%; *cyp51A^PxylP^*: 86.8%). This effect was most pronounced in *cprAcyp51A^biPxylP^*. During simultaneous overexpression of both genes, eburicol levels were reduced to 3.2% and ergosterol levels were increased to 89.2%.

### Overexpression of *cprA* in strains carrying *cyp51A* ^*TR34*^ alleles potentiates resistance to azoles

**Fig 4:**
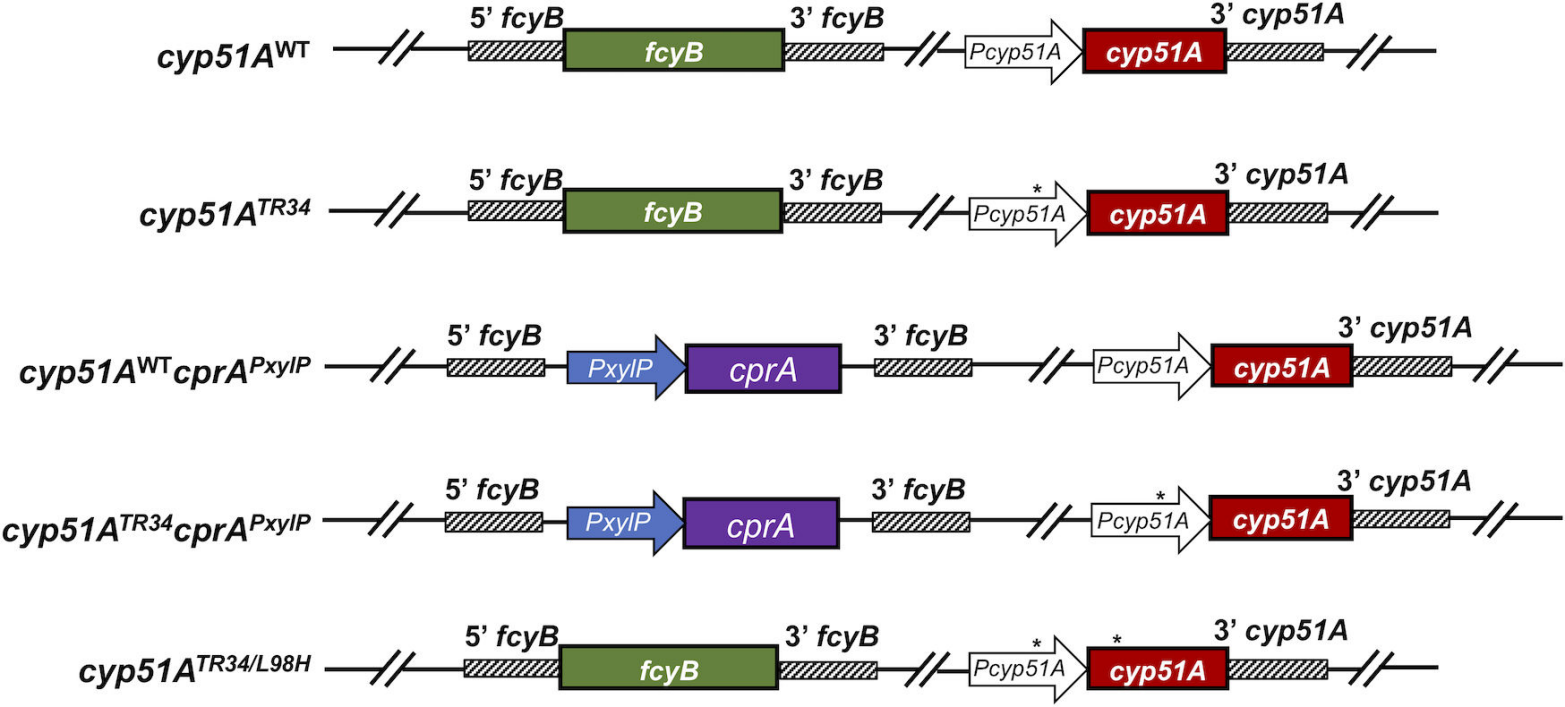
Strains carrying the clinically relevant mutation TR34 and/or the xylose inducible *cprA*. wt and resistance-conferring *cyp51A* alleles were inserted at the *cyp51A* locus in a *cyp51A* deletion background. The inducible *cprA* construct was integrated in *cyp51A* ^WT^ (non-mutated reference allele) and *cyp51A^TR34^* at the *fcyB* locus (36). * denotes TR34 and/or the L98H mutation in the promoter and coding sequence of *cyp51A*, respectively.

Similar to *PxylP*-induced upregulation of *cyp51A*, TR34 leads to elevated *cyp51A* expression and as a result increased azole resistance (13, 15). Our data suggest that additional mutations that elevate CprA activity could further elevate azole resistance in clinical isolates that carry TRs causing increased *cyp51A* expression. To test this idea, a mutant carrying a *cyp51A* allele with the TR34 mutation in its promoter (*cyp51A^TR34^*) was generated and subsequently equipped with the tunable *cprA* expression cassette (*cyp51A^TR34^cprA^PxylP^*, Fig. 4). The strain *cyp51A^TR34/L98H^* harboring the combined *cyp51A* mutation TR34/L98H, resembling one of the most common alleles conferring clinical pan-azole resistance, served as high azole resistance control. As a further reference, strain *cyp51A^WT^* (21) carrying a non-mutated *cyp51A* allele was employed which displayed wt-like azole susceptibilities (Table 2). Depending on the compound tested, *cyp51A^TR34^* and *cyp51A^TR34/L98H^* showed an increase in the MICs of 2- to ≥4-fold and 8- to ≥32-fold, respectively. Similar to *cyp51A^TR34^*, overexpression of *cprA* on its own (*cyp51A^WT^cprA^PxylP^*) increased resistance 2- to 4-fold. In combination with the TR34-allele (*cyp51A^TR34^cprA^PxylP^*), induction of *cprA* elevated MIC levels of the different azoles 8- to ≥32-fold, resembling a similar level of resistance than that observed for *cyp51A^TR34/L98H^*.

**TABLE 2:** MIC values of strains with the clinically relevant mutation TR34 and/or the xylose inducible *cprA^a^*

	VRZ MIC (µg/mL)		ITZ MIC (µg/mL)		ISZ MIC (µg/mL)		TBZ MIC (µg/mL)		EPZ MIC (µg/mL)	
	**−xyl**	**+ xyl**	**−xyl**	**+ xyl**	**−xyl**	**+ xyl**	**−xyl**	**+ xyl**	**−xyl**	**+ xyl**
*cyp51A* ^WT^	0.25	0.25	0.5	0.5	0.5	0.5	1	1	4–8	4–8
*cyp51A^TR34^*	1	1	1–2	1–2	2	1–2	4	4	>16	>16
*cyp51A^WT^cprA^PxylP^*	0.25	1	0.5	1	0.5	1	1	4	4	16
*cyp51A^TR34^cprA^PxylP^*	1	4	1–2	>16	2	4	4	16	>16	>16
*cyp51A^TR34/L98H^*	2–4	2–4	>16	>16	4–8	4	16	16	>16	>16

^*a*^ Voriconazole (VRZ), itraconazole (ITZ), isavuconazole (ISZ), tebuconazole (TBZ), and epoxiconazole (EPZ) susceptibilities of strains were analyzed following the broth microdilution method according to EUCAST (37). Strains were grown in the presence (+xyl) and absence (−xyl) of 1% xylose.

## DISCUSSION

Members of the sterol C14-demethylase Cyp51 family are highly conserved among eukaryotes and one of the best-studied cytochrome P450 enzymes in fungi (38, 39). Cyp51 enzymes in pathogenic fungi are of particular interest for antifungal therapy, as one of the most used antifungal drug classes in clinical treatment, the azoles, target this enzyme (40). The future clinical use of azoles has been challenged over the past years by a dramatic rise in resistance (6, 7). Hence, increasing our knowledge on mechanisms that confer resistance is crucial for the adaptation and optimization of current therapeutic approaches. As stated above, upregulation of *cyp51A* is a key characteristic of TR34/L98H and TR46/Y121F/T289A that contributes to pan-azole resistance (12, 13, 15). In this work, we aimed to elucidate the main redox partner of Cyp51A that might limit azole resistance in *A. fumigatus*. The candidates investigated included CprA and CprB as well as CybE (29, 41). We overexpressed the respective genes together with *cyp51A* using the bidirectional promoter *PxylP* (35) and unveiled CprA as the major factor contributing to Cyp51A-driven azole resistance. Upregulation of *cprA* on its own already led to a moderate increase in resistance to the different azoles tested (2- to 4-fold), which suggests that at least during azole treatment, CprA activity is limiting for Cyp51A activity. The combined overexpression of *cprA* and *cyp51A* elevated MIC levels for all medical azole compounds above the clinical breakpoints suggested by EUCAST (42).

With regard to the distinctive role of CprA in this context, it has to be mentioned that *A. fumigatus* is predicted to express 77 cytochrome P450 enzymes and each CprA, CprB, and CybE might have different specificities for the individual P450 enzymes (29, 41, 43) that have yet to be characterized. Despite the fact that co-overexpression of *cybE* and *cyp51A* did not have a major impact on azole resistance when compared to overexpression of *cyp51A* only (Table 1), previous work demonstrated that the absence of CybE leads to an accumulation of eburicol, a decrease in ergosterol and, in line, increased VRZ susceptibility (29). Moreover, its loss led to a compensatory upregulation of *cyp51A* and *cprA* gene expression (29). Therefore, CybE might not be a rate-limiting factor to elevate azole resistance during *cyp51A* overexpression, but the reduced eburicol turnover in a *cybE* null mutant clearly suggests a crucial role of this component to maintain wt-like sterol C14-demethylation.

The present study suggests that increased activity of the Cyp51A redox partner CprA can further elevate azole resistance during *cyp51A* overexpression. Thus, mutations elevating its activity, e.g., *cprA* promoter mutations or those affecting transcription factors that lead to upregulation of the gene, could cause an unfavorable increase azole resistance during treatment, particularly in strains with preceding mutations that lead to *cyp51A* upregulation such as the abovementioned TRs. In this regard, it is interesting to note that a previously identified clinically resistant isolate carrying TR53, did not contain mutations within the *cyp51A* coding sequence (16). Its level of resistance could not be explained by the TR mutation on its own but rather resulted from extracistronic alterations such as modification of *cprA*. Testing a similar scenario, we overexpressed *cprA* in the strain containing TR34 in the *cyp51A* promoter, which raised resistance to different azoles 4- to ≥8-fold (compare strain *cyp51A^TR34^* with *cyp51A^TR34^cprA^PxylP^*, Table 2).

Collectively, as already indicated in previous work (27, 44 – 46) and further corroborated by this work, we anticipate that inhibitors of CPRs could serve as promising synergistic compounds to counteract azole resistance or to decrease the azole concentrations required for treatment.

## MATERIALS AND METHODS

### Determination of the minimum inhibitory concentration

MIC analyses were performed according to the EUCAST broth microdilution method (37). To induce *PxylP*-driven expression of genes, 1% xylose was supplemented to the medium. Azole compounds used in this study were VRZ, ITZ, ISZ, EPZ, and TBZ (Sigma-Aldrich Corp., St. Louis, MI, USA).

### Generation of plasmids and fungal transformation

Oligonucleotides, strains, and plasmids used in this work are displayed in Tables S1 and S2; Fig. S1, respectively. Generally, to assemble DNA fragments with the plasmid backbones, the NEBuilder HiFi DNA assembly Master Mix (New England Biolabs, Ipswich, MA, USA) was used. Plasmids containing inducible expression cassettes of *cyp51A* only as well as its combination with *cprA*, *cprB,* and *cybE* were constructed as follows. First, the plasmid pΔfcyB_cyp51A*^PxylP^* was generated carrying *cyp51A* under control of *PxylP*. Prior to assembly, the PxylP-*cyp51A* expression cassette was amplified from pΔfcyA_cyp51A*^PxylP^* (47) using primers pX-cass-FW/RV, the backbone allowing integration of the linearized plasmid at the counterselectable marker locus *fcyB* from plasmid pfcyB using primers BBdel-FW/RV (36). To generate bidirectional expression constructs, a backbone was generated by amplifying the plasmid pΔfcyB_cyp51A*^PxylP^* with primers bixylP-BB-FW/RV. *cprA*, *cprB,* and *cybE* coding sequences including 500–800 bp 3′ non-translated region were amplified from genomic DNA employing primers cprAbixylP-FW/RV, cprBbixylP-FW/RV, and cybEbixylP-FW/RV, respectively. Subsequently, the backbone and each component were assembled giving rise to pΔfcyB_cprAcyp51A*^biPxylP^*, pΔfcyB_cprBcyp51A*^biPxylP^,* and pΔfcyB_cybEcyp51A*^biPxylP^*. For single overexpression of *cprA*, the coding sequence was amplified from genomic DNA using cprAxylP-FW/RV. The fragment was then assembled with a backbone amplified from pΔfcyB_cyp51A*^PxylP^* using primers pX-FW.2/RV.2 yielding plasmid pΔfcyB_cprA*^PxylP^*. The plasmid pcyp51A^TR34^, comprising TR34 within the *cyp51A* promoter, was created with the plasmid pcyp51A^WT^ (21) using a DNA duplex. Therefore, 50 µM of each primer 34mer-FW/RV was mixed and annealed by denaturation at 95 °C for 3 min and gradually cooling to room temperature. Subsequently, the duplex was phosphorylated with T4 Polynucleotide kinase (New England Biolabs, Ipswich, MA, USA). Next, the pcyp51A^WT^ plasmid was linearized with cyp51A-TR-BB-FW/RV and ligated with the phosphorylated primer-duplex using T4 DNA ligase (New England Biolabs, Ipswich, MA, USA). The L98H point mutation was introduced into pcyp51A^TR34^ with primers cyp51A-L98H-FW/RV giving rise to pcyp51A^TR34/L98H^.

With the exception of pcyp51A^TR34^ and pcyp51A^TR34/L98H^, plasmids were *Not*I-linearized and transformed into A1160P+ (48), here referred to as wt, resulting in site-directed integrated at the marker locus *fcyB* (47). pcyp51A^TR34^ and pcyp51A^TR34/L98H^ were *Kpn*I-linearized and transformed into ∆*cyp51A*, which led to site-specific integration at the *cyp51A* deletion locus as described recently for pcyp51A^WT^ (21). The resulting strains were designated *cyp51A^TR34^* and *cyp51A^TR34/L98H^. cyp51A^TR34^* and its reference strain *cyp51A^WT^* were further transformed with pΔfcyB_cprA*^PxylP^* giving rise to *cyp51A^TR34^cprA^PxylP^* and *cyp51A^WT^cprA^PxylP^*.

Fungal transformation of plasmids targeting the *fcyB* and *cyp51A* loci was carried out as previously described (21, 36). Correct integrations of constructs were validated by Southern blot analysis.

### Sterol measurements

Sterol analysis of wt, *cyp51A^PxylP^*, *cprA^PxylP^,* and *cprAcyp51A^biPxylP^* was performed in triplicates in AMM (49) containing 1% glucose and 20 mM ammonium tartrate as carbon and nitrogen source, respectively. The xylanase promoter was induced with 1% xylose, and VRZ was used in a final concentration of 0.02 µg/mL. Cultures were inoculated with 1 × 10^6^ spores/mL and incubated at 37 °C for 20 h at 200 rpm. Mycelia were harvested by filtration, shock-frozen, and lyophilized. Subsequently, freeze-dried mycelia were pulverized.

Sterol extraction was performed according to Müller *et al*. (50) using 6 mg freeze-dried mycelia. As described by Müller *et al*. (51), sterols were analyzed as their corresponding trimethylsilyl (TMS) ethers using GC-MS. The individual sterol TMS ethers were identified by their relative retention times (RRT) as well as their specific mass spectra. In total, 12 sterols were detected (for details, see Table S3), and the base peak of each sterol TMS ether was taken as a quantifier ion for calculating the peak areas: cholestane (internal standard, IS) *m/z* 217, RRT 1.00; ergosta-5,8,22-trien-3β-ol (lichesterol) *m/z* 363, RRT 1.29; ergosta-5,7,22-trien-3β-ol (ergosterol) *m/z* 363, RRT 1.32; ergosta-7,22-dien-3β-ol (5-dihydroergosterol) *m/z* 343, RRT 1.34; ergosta-5,7,22,24(28)-tetraen-3β-ol *m/z* 466, RRT 1.35; ergosta-7,22,24(28)-trien-3β-ol *m/z* 343, RRT 1.37; ergosta-5,7,24(28)-trien-3β-ol (5-dehydroepisterol) *m/z* 363, RRT 1.38; ergosta-5,7-dien-3β-ol *m/z* 365, RRT 1.40; ergosta-7,24(28)-dien-3β-ol (episterol) *m/z* 343, RRT 1.40; 4,4,14-trimethylcholesta-8,24(28)-dien-3β-ol (lanosterol) *m/z* 343, RRT 1.43; 4-methylergosta-8,24(28)-dien-3β-ol (4-methylfecosterol) *m/z* 379, RRT 1.45; 4,4,14-trimetylergosta-8,24(28)-dien-3β-ol (eburicol) *m/z* 407, RRT 1.49; 4,4-dimethylergosta-8,24(28)-dien-3β-ol *m/z* 408, RRT 1.51.

For quantification, an external calibration with ergosterol TMS ether was used. The relative sterol levels were determined by plotting the amount of each sterol against the total amount of sterols found in one sample (52).

## SUPPLEMENTAL MATERIAL

Tables S1 to S3 & Fig. S1 aac.00918-23-s0001.pdf The file contains all supplemental Tables and Figures mentioned in the manuscript.

ASM does not own the copyrights to Supplemental Material that may be linked to, or accessed through, an article. The authors have granted ASM a non-exclusive, world-wide license to publish the Supplemental Material files. Please contact the corresponding author directly for reuse.
