# Supplementary material for: The cytochrome P450 reductase CprA is a rate-limiting factor for Cyp51A-mediated azole resistance in Aspergillus fumigatus
Source: Antimicrob Agents Chemother. 2023 Oct 10;67(11):e00918-23. doi: 10.1128/aac.00918-23 (PMC10648939; doi:10.1128/aac.00918-23)
Supplement: Tables S1 to S3 & Fig. S1 — The file contains all supplemental Tables and Figures mentioned in the manuscript. [file aac.00918-23-s0001.pdf]

Supplemental Information

Table S1. **Oligonucleotides used in this study.**

**Primer**	**Sequence 5′ → 3′**
pX-cass-FW	AATCATGGTCATAGCTGTTTCTGATGCGAGCAACAGTATGC
pX-cass-RV	GAGCGGATAACAATTTCACATGAGGGTTGAGTACGAGATTGG
BBdel-FW	TGTGAAATTGTTATCCGCTCACAA
BBdel-RV	AAACAGCTATGACCATGATTACGC
bixylP-BB-FW	TGTCGTCAAGATTGTAGTGTGTGA
bixylP-BB-RV	AAACAGCTATGACCATGATTACGC
pX-FW.2	CCATGGCAGCAGTGATTTCA
pX-RV.2	GGTTGGTTCTTCGAGTCGATG
cprAbixylP-FW	ACACTACAATCTTGACGACAATGGCGCAACTTGACACG
cprAbixylP-RV	AATCATGGTCATAGCTGTTTCCGACTCGTTGTCTCCAGG
cprBbixylP-FW	ACACTACAATCTTGACGACAATGTCGCTTTTCTCTCAATGGAG
cprBbixylP-RV	AATCATGGTCATAGCTGTTTAACGCCTTTGAGACAAACGC
cybEbixylP-FW	ACACTACAATCTTGACGACAATGTCCGCCTCCAAGGAATTC
cybEbixylP-RV	AATCATGGTCATAGCTGTTTCCGCAACGACTACCGAGTTA
cprAxylP-FW	ATCGACTCGAAGAACCAACCATGGCGCAACTTGACACG
cprAxylP-RV	TGAAATCACTGCTGCCATGGTCACGACCAGACATCCTCCT
cyp51A-TR-BB-FW	GAATCACGCGGTCCGGAT
cyp51A-TR-BB-RV	TAGACAACTCTGAAGTGGTGCTG
34mer-FW	GAATCACGCGGTCCGGATGTGTGCTGAGCCGAAT
34mer-RV	ATTCGGCTCAGCACACATCCGGACCGCGTGATTC
cyp51A-L98H-FW	CAAGGATGTCAATGCGGAAGAGG
cyp51A-L98H-RV	CTTCCGCATTGACATCCTTGTGCTTGCCGTTGAGAATAAACTCG

Table S2. **Strains used in this study.**

**Strain**	**Genotype**	**Reference**
A1160P+ (wt)		(1)
cyp51A^{PxylP}	Δ*fcyB::PxylP-cyp51A*	This study
cprA^{PxylP}	Δ*fcyB::PxylP-cprA*	This study
cybEcyp51A^{biPxylP}	Δ*fcyB::cybE-PxylP-cyp51A*	This study
cprAcyp51A^{biPxylP}	Δ*fcyB::cprA-PxylP-cyp51A*	This study
cprBcyp51A^{biPxylP}	Δ*fcyB::cprB-PxylP-cyp51A*	This study
cyp51A^{WT}	Δ*cyp51A::ble, Pcyp51A-cyp51A, hph*	(2)
cyp51A^{TR34}	Δ*cyp51A::ble, Pcyp51A*^{TR34}*-cyp51A, hph*	This study
cyp51A$^{TR34/L98H}$	Δ*cyp51A::ble, Pcyp51A*^{TR34}*-cyp51A*^{L98H}*, hph*	This study
cyp51A^{WT}*cprA*^{PxylP}	Δ*cyp51A::ble, Pcyp51A-cyp51A, hph, ΔfcyB::PxylP-cprA*	This study
cyp51A^{TR34}*cprA*^{PxylP}	Δ*cyp51A::ble, Pcyp51A*^{TR34}*-cyp51A, hph, ΔfcyB::PxylP-cprA*	This study

References

1. Fraczek MG, Bromley M, Buied A, Moore CB, Rajendran R, Rautemaa R, Ramage G, Denning DW, Bowyer P. 2013. The cdr1B efflux transporter is associated with non-cyp51a-mediated itraconazole resistance in Aspergillus fumigatus. Journal of Antimicrobial Chemotherapy 68:1486-1496.
2. Kuhbacher A, Peiffer M, Hortschansky P, Merschak P, Bromley MJ, Haas H, Brakhage AA, Gsaller F. 2022. Azole Resistance-Associated Regulatory Motifs within the Promoter of cyp51A in Aspergillus fumigatus. Microbiol Spectr 10:e0120922.

Table S3. **Sterol composition in wt, $cprA^{PxylP}$, $cyp51A^{PxylP}$ and $cprAcyp51A^{biPxylP}$ grown in liquid cultures during inducing (+xyl) and non-inducing (-xyl) conditions in the presence (+VRZ) and absence (-VRZ) of voriconazole.** **1**, lichesterol; **2**, ergosterol; **3**, 5-dihydroergosterol; **4**, ergosta-5,7,22,24(28)-tetraen-3ß-ol; **5**, ergosta-7,22,24(28)-trien-3β-ol; **6**, 5-dehydroepisterol; **7**, ergosta-5,7-dien-3ß-ol; **8**, episterol; **9**, lanosterol; **10**, 4-methylfecosterol; **11**, eburicol; **12**, 4,4-dimethylergosta-8,24(28)-dien-3ß-ol.

		1	2	3	4	5	6	7	8	9	10	11	12
-xyl/-VRZ	wt	0.5%	92.4%	1.0%	0.3%	1.1%	0.2%	0.2%	1.5%	1.1%	0.4%	0.6%	0.7%
-xyl/-VRZ	$cprA^{PxylP}$	0.5%	92.1%	0.9%	0.3%	1.2%	0.2%	0.2%	1.6%	1.0%	0.4%	0.7%	0.8%
-xyl/-VRZ	$cyp51A^{PxylP}$	0.6%	92.5%	1.0%	0.3%	1.1%	0.2%	0.2%	1.5%	0.7%	0.4%	0.7%	0.8%
-xyl/-VRZ	$cprAcyp51A^{biPxylP}$	0.6%	91.7%	1.0%	0.4%	1.4%	0.2%	0.2%	1.6%	0.8%	0.5%	0.9%	0.9%
+xyl/-VRZ	wt	0.6%	91.9%	0.9%	0.4%	1.1%	0.3%	0.2%	1.5%	1.5%	0.4%	0.7%	0.7%
+xyl/-VRZ	$cprA^{PxylP}$	0.8%	90.6%	0.9%	0.4%	1.3%	0.6%	1.1%	1.7%	1.0%	0.4%	0.7%	0.7%
+xyl/-VRZ	$cyp51A^{PxylP}$	0.6%	93.0%	0.9%	0.4%	1.0%	0.3%	0.2%	1.6%	0.7%	0.4%	0.3%	0.7%
+xyl/-VRZ	$cprAcyp51A^{biPxylP}$	0.8%	91.3%	0.9%	0.4%	1.4%	0.7%	0.6%	1.9%	0.8%	0.3%	0.3%	0.6%
-xyl/+VRZ	wt	0.5%	76.5%	0.5%	0.3%	0.6%	0.1%	0.1%	0.8%	3.1%	0.1%	17.1%	0.2%
-xyl/+VRZ	$cprA^{PxylP}$	0.6%	76.3%	0.5%	0.3%	0.6%	0.1%	0.2%	0.8%	3.1%	0.1%	17.3%	0.2%
-xyl/+VRZ	$cyp51A^{PxylP}$	0.6%	76.0%	0.5%	0.4%	0.6%	0.1%	0.2%	0.8%	3.1%	0.1%	17.5%	0.2%
-xyl/+VRZ	$cprAcyp51A^{biPxylP}$	0.6%	76.2%	0.5%	0.4%	0.5%	0.1%	0.1%	0.7%	3.1%	0.1%	17.6%	0.2%
+xyl/+VRZ	wt	0.5%	77.6%	0.4%	0.4%	0.4%	0.1%	0.1%	0.7%	3.2%	0.1%	16.3%	0.1%
+xyl/+VRZ	$cprA^{PxylP}$	0.8%	82.7%	0.7%	0.4%	0.9%	0.4%	0.6%	1.1%	1.5%	0.2%	10.4%	0.4%
+xyl/+VRZ	$cyp51A^{PxylP}$	0.7%	86.8%	0.8%	0.4%	1.3%	0.2%	0.2%	1.5%	1.4%	0.3%	5.8%	0.7%
+xyl/+VRZ	$cprAcyp51A^{biPxylP}$	0.7%	89.2%	0.9%	0.4%	1.2%	0.3%	0.4%	1.4%	1.2%	0.3%	3.2%	0.7%

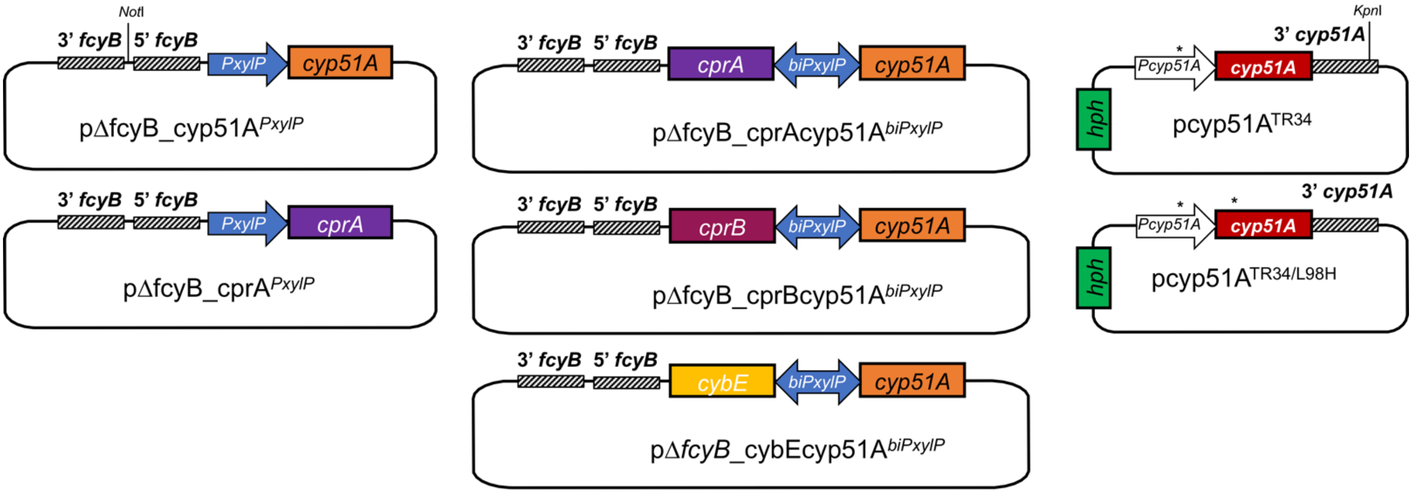

Fig S1. **Scheme of plasmids used in this study.** * denotes TR34 and/or the L98H mutation in the promoter and coding sequence of *cyp51A*, respectively.
